# *Sensors* Best Paper Award 2014

**DOI:** 10.3390/s140101898

**Published:** 2014-01-23

**Authors:** Vittorio M.N. Passaro, W. Rudolf Seitz, Assefa M. Melesse, Alexander Star, Mohamed F. Younis

**Affiliations:** 1 Department of Electrical and Information Engineering, Politecnico di Bari, Via E. Orabona n. 4, 70125 Bari, Italy; E-Mail: passaro@deemail.poliba.it; Tel.: +39-080-5963-850; Fax: +39-080-5963-410; 2 Analytical Chemistry, Department of Chemistry, University of New Hampshire, Durham, NH 03824-3598, USA; E-Mail: wrs@cisunix.unh.edu; Tel.: +1-603-862-2408; Fax: +1-603-862-4278; 3 Department of Earth and Environment, ECS 339, Florida International University, 11200 SW 8th Street Miami, FL 33199, USA; E-Mail: melessea@fiu.edu; Tel.: +1-305-348-6518; Fax: +1-305-348-6137; 4 Department of Chemistry, University of Pittsburgh, 219 Parkman Avenue, Pittsburgh, PA 15260, USA; E-Mail: astar@pitt.edu; Tel.: +1-412-624-6493; Fax: +1-412-624-4027; 5 Department of Computer Science and Electrical Engineering, University of Maryland, Baltimore County, 1000 Hilltop Circle, Baltimore, MD 21250, USA; E-Mail: younis@cs.umbc.edu; Tel.: +1-410-455-3968; Fax: +1-410-455-3969

In 2011, an annual award system was instituted to recognize outstanding *Sensors* papers that are related to sensing technologies and applications and meet the aims, scope and high standards of this journal [1–3]. This year, nominations were made by the Section Editor-in-Chiefs of *Sensors* from among all the papers published in 2010 to track citations. Reviews and full research articles were considered separately. We gladly announce that the following eight papers were awarded the *Sensors* Best Paper Award in 2014.

## Article Award

### 1^st^ Prize

**Heather K. Hunt, Carol Soteropulos and Andrea M. Armani**

Bioconjugation Strategies for Microtoroidal Optical Resonators

*Sensors* **2010**, *10*(10), 9317-9336; doi:10.3390/s101009317

Available online: http://www.mdpi.com/1424-8220/10/10/9317

### 2^nd^ Prize

**Christian H. Schwalb, Christina Grimm, Markus Baranowski, Roland Sachser, Fabrizio Porrati, Heiko Reith, Pintu Das, Jens Müller, Friedemann Völklein, Alexander Kaya and Michael Huth**

A Tunable Strain Sensor Using Nanogranular Metals

*Sensors* **2010**, 10(11), 9847-9856; doi:10.3390/s101109847

Available online: http://www.mdpi.com/1424-8220/10/11/9847

### 3^rd^ Prize

**Rozalia Orghici, Peter Lützow, Jörg Burgmeier, Jan Koch, Helmut Heidrich, Wolfgang Schade, Nina Welschoff and Siegfried Waldvogel**

A Microring Resonator Sensor for Sensitive Detection of 1,3,5-Trinitrotoluene (TNT)

*Sensors* **2010**, *10*(7), 6788-6795; doi:10.3390/s100706788

Available online: http://www.mdpi.com/1424-8220/10/7/6788

### 4^th^ Prize

**Andrea Mannini and Angelo Maria Sabatini**

Machine Learning Methods for Classifying Human Physical Activity from On-Body Accelerometers

*Sensors* **2010**, *10*(2), 1154-1175; doi:10.3390/s100201154

Available online: http://www.mdpi.com/1424-8220/10/2/1154

### 5^th^ Prize

**Martin Nirschl, Arto Rantala, Kari Tukkiniemi, Sanna Auer, Ann-Charlotte Hellgren, Dana Pitzer, Matthias Schreiter and Inger Vikholm-Lundin**

CMOS-Integrated Film Bulk Acoustic Resonators for Label-Free Biosensing

*Sensors* **2010**, *10*(5), 4180-4193; doi:10.3390/s100504180

Available online: http://www.mdpi.com/1424-8220/10/5/4180

## Review Award

### 1^st^ Prize

**Eun-Hyung Yoo and Soo-Youn Lee**

Glucose Biosensors: An Overview of Use in Clinical Practice

*Sensors* **2010**, *10*(5), 4558-4576; doi:10.3390/s100504558

Available online: http://www.mdpi.com/1424-8220/10/5/4558

### 2^nd^ Prize

**Yoshikazu Kobayashi, Masaaki Habara, Hidekazu Ikezazki, Ronggang Chen, Yoshinobu Naito and Kiyoshi Toko**

Advanced Taste Sensors Based on Artificial Lipids with Global Selectivity to Basic Taste Qualities and High Correlation to Sensory Scores

*Sensors* **2010**, *10*(4), 3411-3443; doi:10.3390/s100403411

Available online: http://www.mdpi.com/1424-8220/10/4/3411

### 3^rd^ Prize

**George F. Fine, Leon M. Cavanagh, Ayo Afonja and Russell Binions**

Metal Oxide Semi-Conductor Gas Sensors in Environmental Monitoring

*Sensors* **2010**, *10*(6), 5469-5502; doi:10.3390/s100605469

Available online: http://www.mdpi.com/1424-8220/10/6/5469

The prize awarding committee merits the article “Bioconjugation Strategies for Microtoroidal Optical Resonators” as a “significant experimental work to extend label-free biosensors performance to specificity by surface functionalization (silica microtoroidal case)”. The review “Glucose Biosensors: An Overview of Use in Clinical Practice” “details, in depth, the analytical requirements in terms of precision of measurement, statistical accuracy…; and will “…enable a perspective on the future direction of the medically accepted correlation.”, and further “…moves from the basic principles of these important devices to actual practice”.

These eight exceptional papers are valuable contributions to *Sensors* and the sensing field. On behalf of the Prize Awarding Committee and the Editorial Board of *Sensors*, we would like to congratulate these eight teams for their excellent work. In recognition of their accomplishment, Drs. Andrea M. Armani, Christian H. Schwalb, Rozalia Orghici, Angelo Maria Sabatini and Martin Nirschl will receive 1,000 CHF, 800 CHF, 600 CHF, 400 CHF and 200 CHF, respectively, and the privilege of publishing an additional open access format paper of their choice, free of charge, in *Sensors* in 2014. Drs. Soo-Youn Lee, Yoshikazu Kobayashi, and Russell Binions will be awarded the privilege of publishing an additional research paper free of charge in open access format in *Sensors*.

### Prize Awarding Committee

*Editor-in-Chief*, Section ‘Physical Sensors’

**Dr. Vittorio M.N. Passaro**

Department of Electrical and Information Engineering, Politecnico di Bari, Via E. Orabona n. 4, 70125 Bari, Italy

Tel.: +39-080-5963-850; Fax: +39-080-5963-410

Website: http://dee.poliba.it/photonicsgroup

E-Mail: passaro@deemail.poliba.it

*Editor-in-Chief*, Section ‘Chemical Sensors’

**Prof. Dr. W. Rudolf Seitz**

Analytical Chemistry, Department of Chemistry, University of New Hampshire, Durham, NH 03824-3598, USA

Tel.: +1-603-862-2408; Fax: +1-603-862-4278

Website: http://www.unh.edu/chemistry/faculty/seitz_w.html

E-Mail: wrs@cisunix.unh.edu

*Editor-in-Chief*, Section ‘Remote Sensors’

**Dr. Assefa M. Melesse**

Department of Environmental Studies, ECS 339, Florida International University, 11200 SW 8th Street, Miami, FL 33199, USA

Tel.: +1-305-348-6518; Fax: +1-305-348-6137

Website: http://www.fiu.edu/∼melessea/

E-Mail: assefa.melesse@fiu.edu

*Editor-in-Chief*, Section ‘Biosensors’

**Dr. Alexander Star**

Department of Chemistry, University of Pittsburgh, 219 Parkman Avenue, Pittsburgh, PA 15260, USA

Tel.: +1-412-624-6493; Fax: +1-412-624-4027

Website: http://www.pitt.edu/∼astar/

E-Mail: astar@pitt.edu

*Editor-in-Chief*, Section ‘Sensor Networks’

**Dr. Mohamed F. Younis**

Department of Computer Science and Electrical Engineering, University of Maryland, Baltimore County, 1000 Hilltop Circle, Baltimore, MD 21250, USA

Tel.: +1-410-455-3968; Fax: +1-410-455-3969

Website: http://www.csee.umbc.edu/∼younis

E-Mail: younis@cs.umbc.edu
